# Predictive Value of Baseline FDG-PET/CT for the Durable Response to Immune Checkpoint Inhibition in NSCLC Patients Using the Morphological and Metabolic Features of Primary Tumors

**DOI:** 10.3390/cancers14246095

**Published:** 2022-12-11

**Authors:** Ken Kudura, Nando Ritz, Tim Kutzker, Martin H. K. Hoffmann, Arnoud J. Templeton, Robert Foerster, Michael C. Kreissl, Kwadwo Antwi

**Affiliations:** 1 Department of Nuclear Medicine, Sankt Clara Hospital, 4058 Basel, Switzerland; 2 Faculty of Medicine, University of Basel, 4058 Basel, Switzerland; 3 Faculty of Applied Statistics, Humboldt University, 10 117 Berlin, Germany; 4 Sankt Clara Research, 4002 Basel, Switzerland; 5 Department of Radiooncology, Cantonal Hospital Winterthur, 8400 Winterthur, Switzerland; 6 Division of Nuclear Medicine, Department of Radiology and Nuclear Medicine, University Hospital Magdeburg, 39120 Magdeburg, Germany

**Keywords:** positron emission tomography computed tomography, lung cancer, NSCLC, immunotherapy, CTLA-4, PD-1, PD-L1, outcome prediction

## Abstract

**Simple Summary:**

Lung cancer is the leading cause of cancer-related death worldwide. Nonsmall cell lung cancer (NSCLC) accounts for 80–85% of all cases. Immune checkpoint inhibitors (ICIs) have revolutionized the field of oncology by improving survival in cancer patients. However, given their limited response rate and high immunotoxicity, an accurate selection of NSCLC patients eligible for ICIs appears to be of great importance. We aimed to investigate the predictive value of baseline 2-deoxy-2-[^18^F]fluoro-D-glucose positron emission tomography/computed tomography (FDG-PET/CT) for a durable response to ICIs by linking the morphological and metabolic features of primary tumors in NSCLC patients. The baseline FDG-PET/CT could be used to predict the durable response to ICIs in NSCLC patients. Age, clinical stage IV, lymphangiosis features (on imaging), primary tumor (PT) volume (thus PT metabolic tumor volume MTV due to the demonstrated linear correlation), PT standardized uptake value maximum (SUVmax), and total lesion glycolysis (TLG) were very strong long-term outcome predictors. Our results highlight the importance of linking clinical data, as much as morphological features, to the metabolic parameters of primary tumors in a multivariate outcome-predicting model using baseline FDG-PET/CT.

**Abstract:**

Objectives: We aimed to investigate the predictive value of baseline 2-deoxy-2-[^18^F]fluoro-D-glucose positron emission tomography/computed tomography (FDG-PET/CT) for durable responses to immune checkpoint inhibitors (ICIs) by linking the morphological and metabolic features of primary tumors (PTs) in nonsmall cell lung cancer (NSCLC) patients. Methods: For the purpose of this single-center study, the imaging data of the patients with a first diagnosis of NSCLC and an available baseline FDG-PET/CT between 2020 and 2021 were retrospectively assessed. The baseline characteristics were collected based on clinical reports and interdisciplinary tumor board documentation. The metabolic (such as standardized uptake value SUV maximum and mean (SUV_max_, SUV mean), metabolic tumor volume (MTV), total lesion glycolysis (TLG)) and morphological (such as volume, morphology, margin, and presence of lymphangiosis through imaging) features of all the PTs were retrospectively assessed using FDG-PET/CT. Overall survival (OS), progression-free survival (PFS), clinical benefit (CB) and mortality rate were used as endpoints to define the long-term response to therapy. A backward, stepwise logistic regression analysis was performed in order to define the best model for predicting lasting responses to treatment. Statistical significance was assumed at *p* < 0.05. Results: A total of 125 patients (median age ± standard deviation (SD) 72.0 ± 9.5 years) were enrolled: 64 men (51.2%) and 61 women (48.8%). Adenocarcinoma was by far the most common histological subtype of NSCLC (47.2%). At the initial diagnosis, the vast majority of all the included patients showed either locally advanced disease (34.4%) or metastatic disease (36.8%). Fifty patients were treated with ICIs either as a first-line (20%) or second-line (20%) therapy, while 75 patients did not receive ICIs. The median values ± SD of PT SUV_max_, mean, MTV, and TLG were respectively 10.1 ± 6.0, 6.1 ± 3.5, 13.5 ± 30.7, and 71.4 ± 247.7. The median volume of PT ± SD was 13.7 ± 30.7 cm^3^. The PTs were most frequently solid (86.4%) with irregular margins (76.8%). Furthermore, in one out of five cases, the morphological evidence of lymphangiosis was seen through imaging (n = 25). The median follow-up ± SD was 18.93 ± 6.98 months. The median values ± SD of OS and PFS were, respectively, 14.80 ± 8.68 months and 14.03 ± 9.02 months. Age, PT volume, SUV_max_, TLG, the presence of lymphangiosis features through imaging, and clinical stage IV were very strong long-term outcome predictors of patients treated with ICIs, while no significant outcome predictors could be found for the cohort with no ICI treatment. The optimal cut-off values were determined for PT volume (26.94 cm^3^) and SUVmax (15.05). Finally, 58% of NSCLC patients treated with ICIs had a CB vs. 78.7% of patients in the cohort with no ICI treatment. However, almost all patients treated with ICIs and with disease progression over time died (mortality in the case of disease progression 95% vs. 62.5% in the cohort without ICIs). Conclusion**:** Baseline FDG-PET/CT could be used to predict a durable response to ICIs in NSCLC patients. Age, clinical stage IV, lymphangiosis features through imaging, PT volume (thus PT MTV due to a previously demonstrated linear correlation), PT SUV_max_, and TLG were very strong long-term outcome predictors. Our results highlight the importance of linking clinical data, as much as morphological features, to the metabolic parameters of primary tumors in a multivariate outcome-predicting model using baseline FDG-PET/CT.

## 1. Introduction

Lung cancer is the leading cause of cancer-related death worldwide, responsible for more deaths than prostate, breast and colon cancer taken together. The annual mortality rate of lung cancer is still rising worldwide, with 1.80 million deaths in 2020 [1,2,3,4]. Nonsmall cell lung cancer (NSCLC) accounts for 80–85% of the cases, with small cell lung cancer SCLC contributing the remaining 15–20% [2,5,6]. At diagnosis, more than 60% of NSCLC patients display either a locally advanced or metastatic disease and are therefore no longer eligible for surgical treatment alone, requiring further treatment options [2].

In the last decade, a new class of monoclonal antibodies (immune checkpoint inhibitors (ICIs)) has been introduced into clinical practice and revolutionized the field of oncology, improving survival in many cancer patients. ICIs, such as cytotoxic T lymphocyte-associated molecule-4 (CTLA-4), programmed cell death receptor-1 (PD-1), and programmed cell death ligand-1 (PD-L1), are designed to enhance the systemic antitumor immune response by interrupting coinhibitory signal pathways and eliminating tumor cells [7]. Several cancer types have shown favorable clinical responses to ICIs, including melanoma and NSCLC [8,9,10,11]. However, this groundbreaking treatment approach also has some limitations. In fact, only 20% of advanced NSCLC patients receiving ICIs achieve an objective response; on the other hand, 7–27% experience severe immune-related adverse events (IRAEs) [4,8,9,12,13]. Given their limited response rate and high immunotoxicity, the accurate selection of NSCLC patients eligible for ICIs appears crucial. Therefore, several biomarkers for responses to ICIs have been extensively discussed in the recent literature, such as PD-L1 status and tumor mutational burden (TMB) [4,7,12,13,14].

In the context of NSCLC, PD-L1 expression on tumor cells has been proposed as a preferred biomarker for the prediction of responses to ICIs [3,8,9,12,13]. However, several limitations of this biomarker in clinical practice are known. First of all, a large proportion of NSCLC patients with low or no PD-L1 expression (PD-L1 < 50%) show clinical benefits from ICIs, while others with high PD-L1 expression (≥50%) do not. Secondly, the heterogenous intratumoral PD-L1 expression and variable expression within the tumor microenvironment (TME) renders its use as the only biomarker for response predication to ICIs unsatisfactory [13,15]. Finally, not all advanced NSCLC patients are suitable for biopsy and pathological assays. In the knowledge of these limitations, noninvasive biomarkers are urgently needed to predict response to ICIs [13].

2-deoxy-2-[^18^F]fluoro-D-glucose positron emission tomography/computed tomography (FDG-PET/CT) has been widely used as an integral part of clinical staging in NSCLC patients [16]. Additionally, there has been rising interest in the recently published literature on metabolic parameters derived from FDG-PET/CT to assess the short-term response to ICIs in NSCLC patients with less or no consideration for morphological features [12,15,17,18].

With the knowledge of the relevant recent investigations, we aimed to assess the predictive value of baseline FDG-PET/CT for the prediction of a durable response to ICIs by linking the morphological and metabolic features of primary tumors in NSCLC patients.

## 2. Materials and Methods

### 2.1. Patient Cohort

For the purposes of the following single-center retrospective study, the clinical records of the interdisciplinary tumor center of the Sankt Clara Hospital in Basel (Switzerland) were retrospectively reviewed. Data from all consecutive patients fulfilling the following criteria were analyzed:

(1). The patient was initially diagnosed with a pathologically confirmed NSCLC between 1 January 2020 and 31 December 2021 at the Sankt Clara Hospital in Basel (Switzerland);
(2). The patient underwent an FDG-PET/CT scan performed at the Sankt Clara Hospital in Basel (Switzerland) for staging before any treatment with regards to the proven NSCLC;
(3). The patient was over the age of 18 years at the date of NSCLC diagnosis;
(4). The patient consented to the use of their clinical data for research purposes.

This study was conducted in compliance with good clinical practice (GCP) rules and the Declaration of Helsinki, with the approval of the Northwestern and Central Switzerland ethics committee (Ethikkommission Nordwest- und Zentralschweiz (EKNZ)-Nr: 2022-00248) on 28 March 2022.

### 2.2. Baseline Characteristics

The following clinical data were collected based on medical reports including interdisciplinary tumor board decisions for all the included patients: age (in years), sex (male/female), body mass index (BMI) in kilograms per square meter (kg/m^2^), histopathological subtype of NSCLC (adenocarcinoma, squamous cell carcinoma, large cell carcinoma, and neuroendocrine tumor), PD-L1 expression (in %), number of distant metastasis/-es, anatomical site of distant metastasis/-es (liver, lung, pleura, bone, suprarenal gland, kidney, soft tissue, brain, and lymph node), clinical stage (I-IV) in accordance with the American Joint Committee on Cancer (AJCC) stage (8th edition), and treatment regimen (first-line ICI, second-line ICI, no ICI).

For the purposes of the investigations, patients were dichotomized into two groups: patients treated with ICI (in first- or second-line therapy) vs. patients with no ICI treatment.

### 2.3. FDG-PET/CT Acquisition

All baseline FDG-PET/CT scans considered for the purposes of the investigations were performed in clinical routine at the Department of Nuclear Medicine in the Sankt Clara Hospital in Basel (Switzerland) with one discovery MI PET/CT scanner by General Electric (GE), according to the department’s standard protocol.

Patients were asked to fast at least 4 h prior to intravenous 18F-FDG-administration. A blood glucose level below 10.0 millimoles per liter (mmol/L) was required (mean 6.3 mmol/L, interquartile range (IQR) 5.6–6.6) before the intravenous injection of ^18^F-FDG (mean 265.7 Megabecquerel (MBq)), IQR 203.8–316.6). Image acquisition began 60 min after the administration of a BMI-adapted 18F-FDG dose from the vertex of the skull to the thighs in the supine position. In the absence of renal impairment or allergy, a diagnostic CT scan with iodinated contrast medium and dedicated chest acquisition was performed for attenuation correction and diagnostic purposes (mean dose length product DLP from vertex of skull to mid thighs: 573.4 Milligray per cm (mGy/cm), IQR 355.2–729.0) followed by a three-dimensional (3D) PET acquisition using time-of-flight (TOF) technique.

All baseline FDG-PET/CT scans considered were reported in clinical routine by two physicians (a board-certified radiologist and nuclear physician) in accordance with the department’s standard clinical workflow.

### 2.4. Primary Tumor Segmentation

#### 2.4.1. Metabolic Features

The primary lung tumor was retrospectively delineated on the coregistered CT- and PET-images using a manual 3D-contouring tool at an advanced workstation, General Electrics (GE AW) 4.7. The mean and maximum standardized uptake value (SUV), as well as the metabolic tumor volume (MTV) and total lesion glycolysis (TLG) of each included NSCLC were extracted from the same volume of interest (VOI) surrounding the whole primary tumor on the PET images reconstructed with ordered subset expectation maximization (OSEM) and a threshold set at 42% of the SUVmax.

#### 2.4.2. Morphological Features

The volume of each included primary lung tumor was measured using a VOI surrounding the whole tumor from the CT scans (mostly with iodinated contrast medium). The CT-based contours could be manually corrected by matching the lesion borders on the CT- and PET-images.

Additionally, further morphological features of each included case of NSCLC on the contrast CT scan were reported, such as anatomical site (upper lobe (UL) R: right or L: left; lower lobe (LL) R: right or L: left; middle lobe (ML)), localization within the lobe (central, peripheral, extensive), morphology (solid, subsolid, solid and subsolid/mixed, cystic), margin (sharp, irregular, spiculated), and features of lymphangiosis carcinomatosa from the CT scan (yes/no).

### 2.5. Long-Term Response Assessment

In order to assess the long-term response to treatment, overall survival (OS) and progression-free survival (PFS) were reported based on internal medical reports. OS was defined as the time from the date of NSCLC diagnosis to death or last follow-up, while PFS was defined as the time from the date of NSCLC diagnosis to disease progression (based on imaging and/or clinical findings) or death.

Furthermore, a new variable, clinical benefit (CB), was introduced. CB was defined as no disease progression from treatment initiation to last follow-up.

Finally, the mortality rate from the date of diagnosis to the last follow-up was reported.

All four endpoints for long-term response to therapy (OS, PFS, CB, and mortality rate) were assessed on 19 August 2022.

### 2.6. Statistical Analysis

For descriptive statistical analyses, the median, standard deviation (SD), and IQR were used to characterize the continuous variables. Categorical variables were described using frequencies. A backward, stepwise logistic regression analysis was performed in order to define a strong model predicting the lasting response to treatment. A Cox proportional hazard regression model was used to define the optimal cut-off values for the significant outcome predictors with their hazard ratio (HR) and a 95%-confidence interval (CI). Optimal cut-off values were defined as the values for which the two considered reference groups differed the most in their probability of survival over time. Kaplan-Meier survival curves were then generated with regard to the predefined optimal cut-off values for OS and PFS. Statistical significance was accepted at *p* < 0.05. No adjustment was made for multiple statistical testing. All statistical computations were performed using R (version 4.1.1).

## 3. Results

### 3.1. Baseline Characteristics 

A total 125 patients with proven histopathological NSCLC (median age ± SD 72.0 ± 9.5 years, median BMI ± SD 24.8 ± 5.9 kg/m^2^) were enrolled, applying the above-listed inclusion criteria: 61 were women (48.8%), and 64 were men (51.2%).

Adenocarcinoma was by far the most common histological subtype of NSCLC, followed by squamous cell carcinoma, accounting together for 72% of the cases.

At diagnosis, the majority of all included patients (71.2%, n = 89) showed either locally advanced disease (34.4%, n = 43) or metastatic disease (36.8%, n = 46).

In the case of metastatic disease, bone (21.0%, n = 17), lung (17.3%, n = 14), pleura (16.0%, n = 13), and suprarenal glands (14.8%, n = 12) were the most frequent anatomical sites for distant metastases constituting almost 70% of all of the distant metastases.

Only 19.2% of the cohort (n = 24) showed high PD-L1 expression (>50%), while most of the patients had either low or no PD-L1-expression (1–50%).

A total of 50 patients were treated with ICIs either as first-line (n = 25, 20%) or second-line (n = 25, 20%) therapy, while 75 patients did not receive ICIs in first- or second-line treatment (Table 1).

### 3.2. Primary Tumor Segmentation 

#### 3.2.1. Metabolic Features of Primary Tumor

The median values ± SD for SUV max, mean, MTV, and TLG were, respectively, 10.1 ± 6.0, 6.1 ± 3.5, 13.5 ± 30.7, and 71.4 ± 247.7 (Table 2).

#### 3.2.2. Morphological Features of Primary Tumor

The median tumor volume ± SD was 13.7 ± 30.7 cm^3^. The primary lung tumors were most frequently solid (86.4%, n = 108), with irregular margins (76.8%, n = 96), and were often located in the upper lobes (58.4%, n = 73).

Furthermore, in one out of five cases, morphological evidence for lymphangiosis was seen on CT (n = 25) (Table 2).

### 3.3. Long-Term Response Assessment 

The median follow-up ± SD was 18.93 ± 6.98 months. The median values ± SD for OS and PFS were, respectively, 14.80 ± 8.68 and 14.03 ± 9.02 months.

The majority of all the included patients (70.4%, n = 88) had CB from the treatment with no disease progression; however, 24.0% (n = 30) had deceased during follow-up (Table 3).

### 3.4. Prediction Model for Durable Treatment Response

For the purposes of the investigations, the patients were dichotomized into two groups: patients treated with ICIs in first- or second-line therapy (40%, n = 50) vs. patients with no ICI treatment (60%, n = 75).

A backward, stepwise multivariate logistic regression analysis was chosen as systematic approach to define a strong model predicting lasting response to treatment. However, in order to reduce the so-called “perfect correlation effect”, during the regression analyses, an empiric correlation matrix was generated in the first place. The generated correlation matrix displayed a correlation factor of 0.995 between volume and MTV, suggesting a perfectly linear behavior between volume and MTV. For this reason, MTV was removed from further regression analyses since its properties can be automatically derived from tumor volume properties given their linear correlation.

All the other clinical, metabolic, and morphological variables were initially taken into account for the backward, stepwise multivariate logistic regression analysis. Subsequently, the least significant variables (variables with the highest p values) were removed one after the other until no further variables could be deleted without a statistically significant deterioration of the model.

The multivariate model suggested several clinical, metabolic, and morphological features as strong predictors for lasting responses to ICI.

Table 4 summarizes the results of the backward, stepwise multivariate logistic regression analysis performed after taking all clinical, metabolic, and morphological variables into account in all the included patients. The table displays all the parameters suggested by the model for predicting a lasting response to treatment based on OS and PFS in patients treated with immunotherapy vs. no immunotherapy. Each predictor is listed with its hazard ratio, HR, the corresponding 95%-confidence interval, CI, in brackets below the HR, as much as the significance level right next to HR and CI for each endpoint for the lasting response to treatment, respectively OS and PFS in the patients treated with immunotherapy vs. patients treated with no immunotherapy. In fact, age (HR OS 0.92, *p* < 0.01; HR PFS 0.92, *p* < 0.01), SUVmax (HR OS 1.36, *p* < 0.01; HR PFS 1.30, *p* < 0.01), volume (HR OS 1.07, *p* < 0.01; HR PFS 1.06, *p* < 0.01), TLG (HR OS 0.99, *p* < 0.01; HR PFS 0.99, *p* < 0.01), the presence of lymphangiosis features through imaging (HR OS 4.3, *p* = 0.04; HR PFS 4.53, *p* = 0.03), and clinical stage IV (HR OS 6.13, *p* = 0.04; HR PFS 6.89, *p* = 0.03) were very strong long-term outcome predictors in patients treated with ICIs, while no significant outcome predictors (*p* > 0.05) could be found for patients with no ICI treatment (Table 4).

Given these results, some observations should be highlighted at this point for a better understanding of the following analyses.

First of all, age, as a predictor in our multivariate predicting model presented an HR of <1.00 for OS and PFS, respectively (0.92), which should be questioned. The initially generated correlation matrix provided a statistically well-founded explanation. The correlation index between age and clinical stage IV was strongly positive, e.g., the older the patient the higher the likelihood of clinical stage IV. Given the high HR of clinical stage IV (HR OS and PFS: 6.13 and 6.89), the low HR of age could be compensatory low (HR < 1.00), e.g., should the negative effect of clinical stage IV on OS and PFS be overestimated, the underestimated effect of age might be used as a correction factor in the predicting model. A similar explanation may be applied for TLG with an HR of <1.00 for OS and PFS, respectively, of 0.990 and 0.992 vs. SUVmax, with both having a strongly positive correlation index.

Table 5 summarizes the impact of the predictive parameters described above on long-term outcomes, depending on whether the considered predictive parameter was lower or higher than the predefined cut-off value for the continuous predictive parameters or whether the considered predictive parameter was present or not for the categorical predictive parameter. The cut-off values were determined for the continuous strong predictors for an HR > 1.00 only: volume (26.94 cm^3^) and SUVmax (15.05), respectively, since age and TLG (both HR < 1.00), as previously stated, could be statistically interpreted as correction factors for the strong negative effect of clinical stage and SUVmax for the long-term outcome in the presented multivariate prediction model (Table 5).

**Figure 1:**
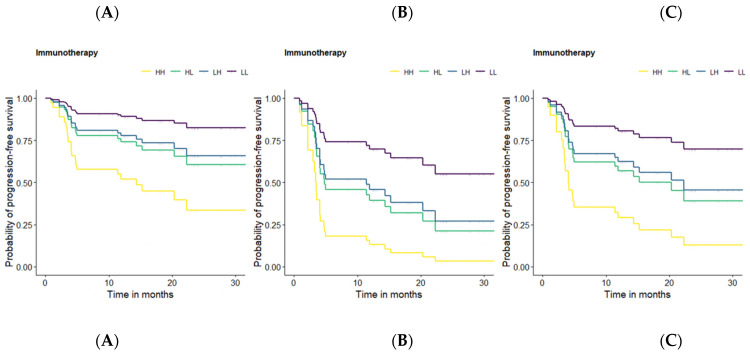

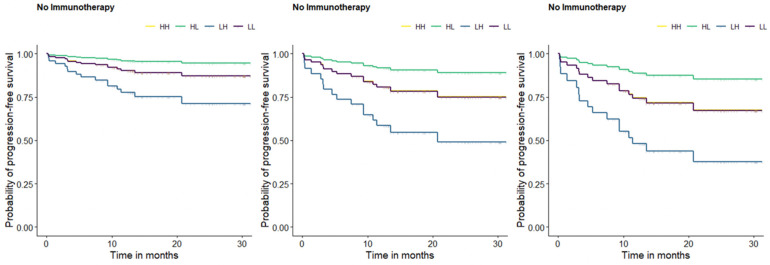
Kaplan-Meier survival curves for PFS based on the defined optimal cut-off values for PT SUVmax, and volume. **HH** = patients with an SUVmax and a volume of primary tumor higher than their respective cut-off-value (yellow). **HL** = patients with a SUVmax higher and a volume of primary tumor lower than the respective cut-off-value (green). **LH** = patients with a SUVmax lower and a volume of primary tumor higher than the respective cut-off-value (blue). **LL** = patients with a SUVmax and a volume of primary tumor lower than their respective cut-off-value (purple). **Survival curve A:** mean age, mean TLG, no lymphangiosis, and clinical stage I-III for all patients. **Survival curve B:** mean age, mean TLG, lymphangiosis, and clinical stage I-III for all patients. **Survival curve C**: mean age, mean TLG, no lymphangiosis, and clinical stage IV for all patients.

Additionally, the presence of lymphangiosis features through imaging and clinical stage IV showed the highest HR for OS and PFS in the patients treated with ICIs and so a strong negative impact on these endpoints over the long-term (after ICI) (Table 4). In order to illustrate this negative impact on the long-term outcomes, Kaplan-Meier survival curves using cut-off values were generated (Figure 1).

Kaplan-Meier survival curves were designed for OS and PFS based on the previously defined optimal cut-off values for the continuous predictors. However, in order to keep a clear presentation, despite numerous display options, we will focus on the PFS curves since no relevant differences were observed between the OS and PFS curves.

Some of the previously presented results should be highlighted at this point for a better understanding of the following figure. Age, SUV*max*, volume, TLG, the presence of lymphangiosis through imaging, and clinical stage IV were suggested by the performed multivariate prediction model as strong predictors for a lasting response to ICIs treatment, with the presence of lymphangiosis through imaging and clinical stage IV having the strongest negative effect on survival. Since age and TLG could be interpretated as correction factors rather than as predictors in the presented prediction model, we aimed at illustrating the negative effect of SUV*max*, volume, the presence of lymphangiosis through imaging, and clinical stage IV using mean age and mean TLG in all the survival curves. For this purpose, a method that enables us to display the influence of the continuous and categorical predictors on PFS in the same figure was required. Therefore, patients with ICI treatment vs. no ICI treatment were divided into four subgroups, depending on whether the considered variable (SUV*max* and volume) was lower or higher than the corresponding predefined cut-off value: HH = patients with a SUVmax and volume of primary tumor higher than their respective cut-off-values (yellow). HL = patients with a SUVmax higher and a volume of primary tumor lower than the respective cut-off-value (green). LH = patients with a SUVmax lower and a volume of primary tumor higher than the respective cut-off-value (blue). LL = patients with a SUVmax and volume of primary tumor lower than their respective cut-off-values (purple). Survival curve A displays the influence of the continuous predictors (SUV*max* and volume) on PFS (in months) without any categorical predictor (lymphangiosis and clinical stage IV). For this reason, survival curve A can be seen as a reference with regard to the influence of the categorical predictors. Survival curve B displays the influence of lymphangiosis through imaging at any clinical stage lower than IV on PFS (in months) in addition to the continuous predictors (SUV*max* and volume). Survival curve C displays the influence of clinical stage IV without lymphangiosis through imaging on PFS (in months) in addition to the continuous predictors (SUV*max* and volume). When comparing survival curves A, B, and C, a significant reduction in the probability of PFS could be observed in survival curves B and C, particularly in survival curve B, highlighting the strong negative effect of lymphangiosis through baseline imaging on long-term survival, as much as clinical stage IV (Figure 1).

**Figure 2:**
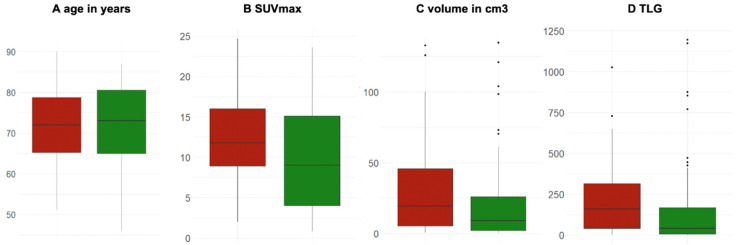
Box plots of the significant predictors for long-term responses in patients treated with ICI (red) vs. no ICI (green). (**A**) Age in years (*p* = 0.64); (**B**) SUVmax unitless (*p* = 0.09); (**C**) volume in cm^3^ (*p* < 0.01), and (**D**) TLG (unitless) (*p* = 0.13).

After testing the prediction power of the baseline FDG-PET/CT in the NSCLC patients with ICI treatment vs. NSCLC patients with no ICI treatment in the first place, we also aimed to investigate whether the predictive biomarkers of the multivariate prediction model showed significant differences between the cohort with ICI (red) vs. no ICI (green) in box plots. Interestingly, only volume was found to have a significant difference between both groups, with a higher mean volume in patients treated with ICI vs. the cohort without ICI (*p* < 0.01). Figure 2

Finally, the long-term response to treatment was further investigated using mortality rate and clinical benefit as endpoints, with interesting results.

**Figure 3:**
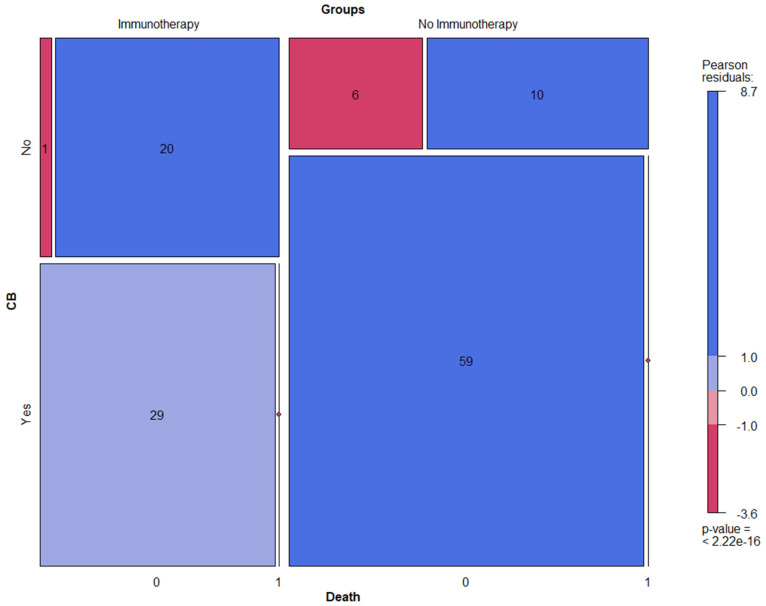
Mosaic plots displaying clinical benefit (CB) and mortality rate in NSCLC patients treated with ICI vs. no ICI.

In fact, 58% of NSCLC patients treated with ICI (n = 29) showed CB vs. 78.7% (n = 59) in the cohort with no ICI treatment. However, the main and highly clinically relevant observation to be highlighted here is that almost all patients treated with ICI and disease progression deceased on follow-up (mortality rate in the case of disease progression was 95% vs. 62.5% in the cohort without ICI) (Figure 3).

## 4. Discussion

We aimed to investigate the predictive value of baseline FDG-PET/CT for the prediction of the durable response to ICIs by linking the morphological and metabolic features of primary tumors in NSCLC patients.

By applying our inclusion criteria, 125 patients with a first diagnosis of NSCLC and an available baseline FDG-PET/CT were retrospectively enrolled, with 64 men and 61 women. The initial cohort was then dichotomized into two groups: 50 patients treated with ICI in first- or second-line therapy vs. 75 patients who did not receive ICI.

The morphological and metabolic features of all the included primary tumors were assessed through coregistered CT- and PET-images.

A backward, stepwise multivariate logistic regression analysis, including patient clinical data and the morphological and metabolic parameters of primary tumors, was chosen to represent a systematic approach for defining a strong model to predict lasting responses to treatment using OS and PFS as long-term endpoints.

Very interesting and potentially clinically relevant results were found, which are now further discussed.

First of all, a multivariate prediction model with strong prediction power was generated for patients treated with ICI, while no significant predictors for long-term outcomes were found in patients with no ICI treatment. In fact, age, primary tumor volume, SUVmax, TLG, the presence of lymphangiosis features through imaging, and clinical stage IV were very strong long-term outcome predictors in those patients treated with ICI. In addition, the optimal cut-off values were determined for primary tumor volume (26.94 cm^3^) and SUVmax (15.05). In light of these results, three relevant insights with regard to the generated prediction model could be emphasized.

First, the initially performed correlation matrix suggested a perfectly linear behavior between primary tumor volume and primary tumor MTV, partly explained by the definition of MTV, e.g., metabolically active volume within a segmented tumor on FDG-PET/CT [19]. Therefore, in order to reduce the so-called “perfect correlation effect” in a multivariate predicting model, only one of the two variables was considered for further regression analyses. Since the purpose of these investigations was to assess the morphological and metabolic features of primary tumors, tumor volume (alongside all the remaining metabolic parameters) was chosen over MTV in the multivariate predicting model, and the MTV properties were subsequently derived automatically from the tumor volume property. Therefore, special caution applies to the “perfect correlation effect” using multivariate regression analyses for outcome prediction.

Secondly, the low HR (<1.00) of age and TLG in the multivariate predicting model suggested that the influence of these two parameters on responses to ICIs might be underestimated in the generated model in order to compensate for the potentially overestimated negative effect on responses to ICIs by clinical stage IV and SUVmax, given their strongly positive indexes. These insights highlighted the importance of questioning the results of the generated multivariate predicting model considering the correlation matrix in order to differentiate the strong predictive parameters from those also acting as correction factors in the model.

Finally, among the four strong predictive biomarkers suggested by the model, firstly, the lymphangiosis on baseline hybrid imaging and then clinical stage IV had the strongest negative impact on long-term response to ICIs, followed by primary tumor volume and SUVmax. These results underlined the importance of linking clinical data, as much as morphological features, to the metabolic parameters of primary tumors, thus revealing the innovative character of the presented multivariate predicting model.

Interestingly, no significant differences were observed regarding patient age, primary tumor SUVmax and TLG between the patients treated with ICI vs. patients treated with no ICI. However, primary tumor volumes at baseline were significantly higher in the patients treated with ICIs compared to those who did not receive ICIs, which might be explained by the higher proportion of patients in stage IV in the cohort treated with ICIs. Nevertheless, these observations underscored the importance of a multivariable approach for outcome prediction since, when taken separately, most of predictive parameters did not show significant differences between the patients treated either with ICIs or no ICIs.

In the long-term, the patient cohort with no ICI treatment showed significantly better clinical benefits and lower mortality (also in case of disease progression) compared to patients treated with ICIs. This could be explained by the composition of the different cohorts rather than the effect of the treatment. In fact, every second patient in the cohort without ICI treatment was at stage I (38.7%) or II (9.3%), while 36% were at stage III and 16% were at stage IV. Different treatment regimens (such as tumor resection or radiotherapy only, tumor resection with platinum-based adjuvant systemic treatment, and platinum-based neoadjuvant systemic treatment followed by tumor resection or radiochemotherapy) were proposed to these patients in accordance with their clinical stage and international guidelines. On the other hand, the patients who received ICI treatment in first- or second-line therapy were exclusively at either stage III (42%) or IV (58%).

Thirteen original manuscripts have been published between 2020 and 2022 on the predictive value of FDG-PET/CT in NSCLC patients, along with a meta-analysis [4,12,15,18,20,21,22,23,24,25,26,27,28,29]. Our study is different in many aspects from these previously published data.

First of all, the main innovative aspect of the present study is the detailed assessment of the morphological features of primary tumors in addition to the metabolic parameters in baseline hybrid imaging alongside clinical data, while most of the previous investigations focused on baseline metabolic parameters only. Our results highlight the importance of linking clinical data, as much as morphological features, to the metabolic parameters of primary tumors in a multivariate outcome-predicting model using baseline FDG-PET/CT.

Secondly, in a recently published meta-analysis on the predictive value of FDG-PET/CT in NSCLC, Zhu et al. included patients in various recent publications with a mean follow-up of 11.9 months, while our mean observation time was longer: 18.9 months, presumably improving the assessment of the prediction power of the generated multivariate prediction model in the long-term. Additionally, our number of patients treated with ICIs was comparable to the average cohort size of those in recent relevant publications on similar topics [4]. Finally, all the effects of the included outcome predictors emerging from our prediction model were all statistically highly significant in the patients treated with ICIs.

However, some limitations of the investigations should also be addressed.

First of all, even though patient baseline characteristics were in accordance with the well-established literature on NSCLC, the retrospective design of this single-center study might have resulted in a more heterogenous cohort (e.g., first- vs. second-line therapy). Prospective studies with larger homogenous populations might be needed to overcome this limitation.

Secondly, the primary tumors were not delineated automatically and not in accordance with a standardized method but were delineated manually through hybrid imaging and so, theoretically, with a certain inaccuracy. However, given the high median volume of all the included primary tumors, as much as the use of contrast medium, this limitation might not have played a significant role in the presented results.

Finally, for the purposes of the investigation, we focused exclusively on the morphological and metabolic features of primary tumors in NSCLC patients. The use of primary tumor metabolic parameters only to predict the response to radiotherapy or other treatment regimens in the context of NSCLC has already been widely discussed and validated by meta-analyses published a few years ago [30,31]. Nevertheless, there has been rising interest in the recent literature on tumor heterogeneity within primary tumors, their environment, and their metastases features in the context of NSCLC. Therefore, the impact of total tumor burden (including metastases features) on the long-term response in NSCLC patients will be extensively discussed in a separate study.

## 5. Conclusions

Baseline FDG-PET/CT could be used to predict the durable response to ICIs in NSCLC patients. Age, clinical stage IV, lymphangiosis features through imaging, PT volume (thus PT MTV due to the previously demonstrated linear correlation), PT SUVmax, and TLG were very strong long-term outcome predictors. Our results highlight the importance of linking clinical data, as much as morphological features, to the metabolic parameters of primary tumors in a multivariate outcome-predicting model using baseline FDG-PET/CT.

## Figures and Tables

**Table 1:** Baseline characteristics of all the included NSCLC patients (N = 125) before initiating treatment.

1. Baseline Characteristics	
**Age median ± SD in years (IQR)**	72.0 ± 9.5 (65.0–80.0)
**Gender**	
Male	64 (51.2%)
Female	61 (48.8%)
**BMI median ± SD in kg/m^2^ (IQR)**	24.8 ± 5.9 (22.3–29.0)
**Histopathological subtype of NSCLC**	
Adenocarcinoma	59 (47.2%)
Squamous cell carcinoma	31 (24.8%)
Large cell carcinoma	17 (13.6%)
Neuroendocrine tumor	15 (12.0%)
Not specific	3 (2.4%)
**PD-L1 expression**	
>50%	24 (19.2%)
1–50%	18 (14.4%)
<1%	57 (45.6%)
Not available	26 (20.8%)
**Distant metastases**	
**On a patient level, in total**	125 (100%)
No distant metastasis	79 (63.2%)
One distant metastasis	33 (26.4%)
Two or more distant metastases	13 (10.4%)
**On a metastasis level, in total**	81 (100%)
Liver	8 (9.9%)
Lung	14 (17.3%)
Pleura	13 (16.0%)
Bone	17 (21.0%)
Suprarenal gland	12 (14.8%)
Kidney	1 (1.2%)
Soft tissue	8 (9.9%)
Brain	1 (1.2%)
Lymph node	7 (8.6%)
**Clinical staging (AJCC 8th edition)**	
I	29 (23.2%)
II	7 (5.6%)
III	43 (34.4%)
IV	46 (36.8%)
**Treatment regimen**	
First-line ICI	25 (20.0%)
Second-line ICI	25 (20.0%)
No treatment with ICI	75 (60.0%)

**Table 2:** Metabolic and morphological features of all segmented NSCLC (N = 125) before initiating treatment.

2. Primary Tumor Segmentation	
**a. Metabolic features of primary tumor**	
SUVmax median ± SD (IQR)	10.1 ± 6.0 (5.8–15.4)
SUVmean median ± SD (IQR)	6.1 ± 3.5 (3.4–8.7)
MTV median ± SD (IQR)	13.5 ± 30.7 (3.5–36.0)
TLG median ± SD (IQR)	71.4 ± 247.7 (14.9–275.1)
**b. Morphological features of primary tumor**	
**Volume median ± SD in cm^3^ (IQR)**	13.7 ± 30.7 (3.5–35.8)
**Anatomical site (lung lobe UL/LL/ML and side R/L)**	
ULR	41 (32.8%)
LLR	21 (16.8%)
ML	14 (11.2%)
ULL	32 (25.6%)
LLL	13 (10.4%)
Two lobes	4 (3.2%)
**Localization of primary tumor within the lobe lung**	
Central	59 (47.2%)
Peripheral	56 (44.8%)
Extensive (central & peripheral)	10 (8.0%)
**Morphology**	
Solid	108 (86.4%)
Subsolid	7 (5.6%)
Mixed (solid/subsolid)	8 (6.4%)
Cystic	2 (1.6%)
**Margin**	
Sharp	13 (10.4%)
Irregular	96 (76.8%)
Spiculated	16 (12.8%)
**Lymphangiosis carcinomatosa**	
Yes	25 (20.0%)
No	100 (80.0%)

**Table 3:** Endpoints used to assess long-term response to treatment.

3.Long-Term Response Assessment	
Follow-up median ± SD in months (IQR)	18.93 ± 6.98 (13.37–25.97)
OS median ± SD in months (IQR)	14.80 ± 8.68 (10.73–23.93)
PFS median ± SD in months (IQR)	14.03 ± 9.02 (9.37–23.60)
CB	
Yes	88 (70.4%)
No	37 (29.6%)
Death	
Yes	30 (24.0%)
No	95 (76.0%)

**Table 4:** The table displays all parameters suggested by the model that predict the lasting response to treatment based on overall survival (OS) and progression-free survival (PFS) in patients treated with immunotherapy vs. no immunotherapy. Each predictor is listed with its hazard ratio, HR, the corresponding 95%-confidence interval, CI, in the brackets below the HR, as much as the significance level right next to HR and CI for each endpoint for the lasting response to treatment, respectively, OS and PFS in patients treated with immunotherapy vs. patients treated with no immunotherapy.

	Immunotherapy				No Immunotherapy			
	OS		PFS		OS		PFS	
	HR (CI)	*p*-Value	HR (CI)	*p*-Value	HR (CI)	*p*-Value	HR (CI)	*p*-Value
Age	0.92 (0.86, 0.98)	<0.01	0.92 (0.86, 0.98)	0.01	1.02 (0.96, 1.09)	0.49	1.03 (0.97, 1.10)	0.28
SUVmax	1.36 (1.14, 1.64)	<0.01	1.30 (1.10, 1.54)	<0.01	1.05 (0.92, 1.19)	0.45	1.08 (0.98, 1.24)	0.23
Volume	1.07 (1.03, 1.12)	<0.01	1.06 (1.02–1.11)	<0.01	1.05 (0.99, 1.11)	0.11	1.04 (1.00, 1.09)	0.06
TLG	0.99 (0.98, 1.00)	<0.01	0.99 (0.99, 1.00	<0.01	0.99 (0.99, 1.00)	0.20	0.99 (0.99, 1.00)	0.09
Lymphangiosis	4.03 (1.06, 17.51)	0.04	4.53 (1.13, 18.20)	0.03	1.70 (0.43 6.71)	0.45	1.69 (0.45, 6.30)	0.44
Stage IV	6.13 (1.10, 34.20)	0.04	6.89 (1.20, 39.56)	0.03	3.87 (0.72, 20.69)	0.11	4.07 (0.84, 19.69)	0.08

**Table 5:** The table summarizes the impact of the predictive parameters described above on the long-term outcome (overall survival (OS) in months and progression-free survival (PFS) in months), depending on whether the considered predictive parameter was lower or higher than the predefined cut-off value for the continuous predictive parameters or whether the considered predictive parameter was present or not for the categorical predictive parameter. For the continuous predictors, such as SUVmax and volume cut-off values were defined for both treatment regimens, e.g., treated with and without immunotherapy. The cut-off values were defined as the values for which the two considered reference groups (≤ vs. >cut-off value) differed the most in their probability of survival over time. For the categorical predictors, such as lymphangiosis and clinical stage IV, the presence of the considered predictor was documented as yes, and the absence as no.

	Immunotherapy				No Immunotherapy			
	OS		PFS		OS		PFS	
SUVmax	≤15.05 15.60	>15.05 13.54	≤15.05 14.95	>15.05 13.11	≤14.36 17.57	>14.36 17.44	≤14.36 17.20	>14.36 14.77
Volume	≤26.94 16.46	>26.94 13.15	≤26.94 15.94	>26.94 12.45	≤24.24 18.34	>24.24 15.31	≤24.24 17.12	>24.24 14.98
Lymphangiosis	No 15.32	Yes 14.40	No 14.70	Yes 13.89	No 18.27	Yes 13.21	No 17.37	Yes 11.82
Clinical stage IV	No 17.69	Yes 13.16	No 16.62	Yes 12.91	No 18.45	Yes 12.72	No 17.39	Yes 12.17

## Data Availability

The data presented in this study are available on request from the corresponding author.

## List of Abbreviations (In Order of Appearance)

NSCLC	Nonsmall cell lung cancer
SCLC	Small cell lung cancer
ICI	Immune checkpoint inhibitors
CTLA-4	Cytotoxic T lymphocyte-associated molecule-4
PD-1	Programmed cell death receptor-1
PD-L	Programmed cell death ligand-1
IRAEs	Immune-related adverse events
TMB	Tumor mutational burden
TME	Tumor microenvironment
FDG-PET/CT	2-deoxy-2-[^18^F]fluoro-D-glucose positron emission tomography/computed tomography
GCP	Good clinical practice
EKNZ	Ethikkommission Nordwest- und Zentralschweiz
BMI	Body mass index
kg/m^2^	Kilograms per squared meter
AJCC	American Joint Committee on Cancer
GE	General Electric
mmol/L	Millimoles per liter
IQR	Interquantile range
MBq	Megabecquerel
DLP	Dose length product
mGy/cm	Milligray per cm
3D	Three-dimensional
TOF	Time-of-flight
AW	Advanced workstation
SUVmax	Standardized uptake value maximum
SUVmean	Standardized uptake value mean
MTV	Metabolic tumor volume
TLG	Total lesion glycolysis
VOI	Volume of interest
OSEM	Ordered subset expectation maximization
UL	Upper lobe
LL	Lower lobe
ML	Middle lobe
OS	Overall survival
PFS	Progression-free survival
CB	Clinical benefit
SD	Standard deviation
HR	Hazard ratio
CI	Confidence interval
